# Systemic Mechanisms of Brain Aging and Rejuvenation

**DOI:** 10.1002/alz.083659

**Published:** 2025-01-03

**Authors:** Saul Villeda

**Affiliations:** ^1^ UCSF Bakar Aging Research Institute, San Francisco, CA USA

## Abstract

**Background:**

Aging drives cellular and cognitive impairments in the adult brain. It is imperative to gain mechanistic insight into what drives aging phenotypes in the brain in order to maintain, and even restore, functional integrity in the elderly.

**Method:**

We, and others, have shown that systemic interventions ‐ such as heterochronic parabiosis (in which a young and old circulatory system are joined) and administration of young blood or exercise induced blood factors ‐ can reverse age‐related impairments in regenerative, synaptic and inflammatory processes, as well as rescue cognitive faculties in the aged brain.

**Result:**

Rejuvenating systemic intervention studies have revealed an age‐dependent bi‐directionality in the influence of the systemic environment indicating pro‐youthful trophic factors in young blood elicit rejuvenation while pro‐aging immune factors in old blood drive aging.

**Conclusion:**

It has been proposed that introducing pro‐youthful trophic factors or mitigating the effect of pro‐aging immune factors may provide effective strategies to rejuvenate aging phenotypes in the brain. Despite this potential, much is unknown as to the systemic and molecular mechanisms regulating the effects of blood‐borne factors. I will discuss work from my research group that begins to provide insight into the beneficial effects of targeting individual systemic immune factors and the downstream molecular drivers promoting rejuvenation in the aging brain.

